# Introductory Lectures

**Published:** 1847-01

**Authors:** 


### INTRODUCTORY LECTURES.

Our thirty medical schools are now in full oparation, and, probably, since the first of November, twice thirty introductory lectures have been published. We have received three of these annuals, which would do credit to any teachers in any medical school. The one which we received first, is from the pen of Professor Caldwell, who, at his advanced age, is toiling on in his profession with all the ardor and earnestness which characterized him when he came to Kentucky nearly thirty years ago. His lecture is divided between advice to his pupils, and a eulogy on the Medical Institute. The former is seasonable and judicious, and the latter, we have no doubt, was sincere. If any of his readers should deem it overstrained, they must remember that the Medical Institute was the child of the author's old age. He had some cause to feel enthusiastic, in view of what that school was, and what he had done in rearing and sustaining it.

The other two lectures are from Philadelphia—one delivered by Professor Hodge, the other by Professor Jackson, of the University of Pennsylvania. Dr. Hodge discourses of obstetrics, the department assigned to him in the University. It is a very appropriate and a very instructive address.

Dr. Jackson's lecture is admirable in matter and manner; its sentiments are elevated, and they are conveyed in a style at once polished and forcible. He speaks of the dignity, aim, tendencies, and dangers of the medical profession, of which we may say, as good old Isaac Walton said of his favorite sport:

> Doubt not, scholar, but that angling is an art, and an art worth your learning; the question is rather whether you be capable of learning it? for angling is like poetry, men are to be born so: I mean with inclinations to it, though both may be heightened by discourse and practice; but he that hopes to be a good angler, must not only have an inquiring, searching, observing wit; but he must bring a large share of hope and patience, and a love and propensity to the art itself.

How the overflowing tide of quackery is to be resisted, Dr. Jackson indicates in the following paragraph:

> Amidst the general decadence that threatens the medical profession, and disrepute overshadowing medical honors, it will be incumbent on the schools of highest reputation, that they should maintain a firm ground. Let them urge on the cultivation of medicine to its highest point as a science, by full and adequate courses. Let them convince the students that it is not for their interests that medicine should be slightingly taught, light as a school boy's task, but must be labored out by application and hard study, through scientific and philosophical instruction. Let them stand firm to their diplomas, and refuse them to the incompetent, and when not fairly earned after a close scrutiny.

